# Construction of the “Full Path” of restorative effects on older adults’ mental health in parks under seasonal differences: taking Changchun as an example

**DOI:** 10.3389/fpubh.2024.1269249

**Published:** 2024-04-09

**Authors:** Tianjiao Yan, Hong Leng, Ziqing Yuan

**Affiliations:** ^1^School of Architecture and Urban Planning, Jilin Jianzhu University, Changchun, China; ^2^Urban Spatial Performance Assessment and Visualization and Decision-Making Lab, Jilin Jianzhu University, Changchun, China; ^3^Research Center for Innovation Development Strategy on the Ecological Wisdom of Urban and Rural Planning in Jilin Province, Changchun, China; ^4^School of Architecture and Design, Harbin Institute of Technology, Harbin, China; ^5^Key Laboratory of National Territory and Spatial Planning and Ecological Restoration in Cold Regions, Ministry of Natural Resources, Harbin, China; ^6^Department of Geography, Faculty of Social and Historical Sciences, University College London, London, United Kingdom

**Keywords:** full path, restorative effects, older adults’ mental health, parks, seasonal differences

## Abstract

With the aging and older adults’ mental health problems in China, more attention has been paid to the restorative environment. As an important restorative environment in the city, the mental health restorative effect of park environment has been confirmed. However, further exploration is needed to determine whether winter parks have positive effects, their differences from non-winter parks, and the specific pathways of these effects. Therefore, this study constructed a “full path” for the restorative effects of older adults’ mental health in parks under seasonal differences, including four components: perceived environment, affective feedback, behavioral feedback, and restorative effect, forming four pathways. Based on this, this study obtained 211 and 240 sample data in winter and non-winter parks, and verified the validity of various hypotheses and mediation paths using structural equation models. It found that: (1) overall restorative effects existed in different seasons; (2) in winter, perceived environmental assessment was not a direct antecedent of restorative effects, and affective feedback and Moderate and Vigorous Physical Activity (MVPA) feedback were important mediating factors, and the chain mediated pathway existed; (3) in non-winter, both direct, indirect and chain mediated effects existed, and affective feedback and Low Physical Activity (LPA) feedback were important mediating factors. Based on this, this study divided parks into “affective inducing” and “behavioral promoting” types, and proposed corresponding planning priorities to positively intervene in planning and design practices.

## 1 Introduction

China is entering an aging society at the fastest pace in history and with a large population of older adults. By 2027, China will transform from an “aging” society to an “aged” society. At the same time, the health challenges faced by older adults in China require more attention. A report showed that nearly one-third of the older adults in China suffer from depression, and the overall prevalence of mild cognitive impairment (MCI) among people aged above 65 is 20.8% (1). Based on this, it can be seen that the situation of aging and mental health issues in China is not optimistic. In 2022, 15 departments including the China Health Commission jointly issued the “14th Five-Year Plan for Healthy Aging,” emphasizing the need to optimize the social environment for healthy living of older adults, better meet their health needs, improve their health level, and extend their healthy life expectancy.

Urban parks have been proven to be one of the main environments for older adults’ activities (2, 3), and their health restorative effects have been widely recognized both domestically and internationally. With the expansion of the dimension of health recovery, the restorative effect of mental health has received more attention. The restorative effects of mental health in parks include both long-term and short-term effects. For one thing, people living near parks and frequently using parks have lower levels of mental stress (4, 5). For the other, 30 min after entering the park, the visitor’s depressive mood is significantly improved (6). The longer they spend in the park, the better their mental state is restored, including restoring attention levels, improving emotional states, enhancing self-worth, and subjective well-being (7–10). Frequent access to the short-term recovery effect of parks can also reduce the risk of mental illness (11). The above effects can have a certain positive guidance on residents’ lifestyles, but for urban planning practitioners, how to implement them in design practice still seems a bit confusing. Therefore, it is even more important to effectively decompose the effect process, positively intervene in the effect path, and implement it in spatial characteristics and elements.

The existing paths of park restorative effects can be roughly divided into two types: direct effects and indirect effects. On the one hand, the former is a direct relationship between the establishment of park environment and restorative effects. Kaplan et al. found that park natural landscape and waterscape have the greatest impact on mental health recovery, and their scale, layout and types have significant differences in the impact of restorative effects (12, 13). Ulrich et al.’s research has shown that people in a suppressed state can significantly alleviate their psychological stress after viewing the park environment (14, 15). These two results are also corresponding to attention recovery theory (ART) and stress relief theory (SRT) in restorative environment. Some studies also focused on the extraction of spatial features and the summary of spatial perception, which can be understood as subjective perception evaluation induced by physical environmental, namely “perceived environment.” For example, some studies divide spatial feature factors into natural factors, perceptual factors, activity factors, and resting factors based on the constituent elements of plants, water bodies, and facilities (16, 17).

On the other hand, indirect effects are the restorative effects exerted by park features through intermediary pathways, which can be roughly divided into discussions on “affection” and “behavior.” One is the discussion involving environmental preferences and place attachment, both of which are individual affect expressions of the environment, hence collectively referred to as “affective feedback.” This mainly includes a simple impact path of “environmental preference → restorative effect” and a composite impact path of “environmental preference → place attachment → restorative effect.” Environmental preference can be understood as an individual’s willingness to choose a certain environment (18), and its correlation with restorative assessment has been proven by many studies, and it has been found that the higher the preference, the stronger the restorative effect of the environment (9). Place attachment describes the emotional relationship between people and places, and people give value to the place through the stable accumulation of emotions (19). Geographers usually believe that individuals need to gain a sense of belonging, self-esteem, and security through their attachment to the place (20), which is related to health and well-being (21, 22). The higher the attachment to the environment, the higher its restorative effect (23). Meanwhile, according to Biophilia Hypothesis, humans are inherently inclined to focus their attention on life or its processes (24), instinctively generating demands or preferences for nature, thereby exhibiting adaptive attachment. Some studies have also confirmed the relationship between environmental preference and place attachment in forests, parks, and natural recreational areas (25, 26). It is worth emphasizing that older residents are more likely to be attached to green spaces than young people. This may be related to the fact that older adults have less mobility than young people and have more time to visit nearby green spaces (27). This is also an important reason for incorporating affective feedback into this study.

For another, there was more research on the discussion of “behavior” mediator. It has shown that as people age, regular exercise can prevent frailty in old age by enhancing muscle strength and quality, as well as maintaining bone density, independence, and vitality (28). Appropriate exercise is associated with better quality of life, positive emotions, and mental health levels (29). In the discussion of behavior patterns, the focus was on the classification of behavior types. Behaviors in parks can be divided into passive, active, and mixed behaviors based on spontaneity (30); necessary activities, spontaneous activities, and social activities according to the content (31); low, medium, and high intensity behaviors according to the intensity of exercise (32). Behavior, as feedback of environmental perception, can directly affect restorative effects. At the same time, the exploration of indirect pathways is also related to the “Cognition-Affect-Behavior” process, which is the ABC attitude model. This model believes that cognition (C) is the antecedent of emotion (A) and behavior (B), and behavior (B) is the decision result of cognition (C) and emotion (A) (33), commonly seen in consumer behavior and psychological therapy. In the field covered by this article, the park environment can be regarded as a service product, while the perceived environmental evaluation is a cognitive result, and the visitors’ feelings, decision-making, and recovery processes in it are similar. This can help research establish the relationship between affect and behavior.

In addition, existing research focused more on non-winter parks, and although some studies have now shifted their focus to winter, it still appeared insufficient. Especially, older adults in cold regions are affected by extreme weather conditions and exhibit more significant depressive and seasonal emotional problems. Therefore, the discussion of winter in cold regions is also of great significance. Some studies have shown that plant landscapes covered with snow in winter exhibit different visual effects and may also have a restorative effect (34). Winter and summer also have attention recovery effects, and seasonal plant visual changes can enhance the recovery effect (35). However, most of these studies focused on young people and were conducted through VR experiments. Laboratory experiments cannot truly reflect individuals’ feelings in the environment and have certain limitations.

Overall, there are three issues with relevant research: Firstly, most existing research explored a single path from a certain perspective, and mostly discussed behavior, lacking a detailed breakdown of psychological processes and a comprehensive grasp of the entire path. Secondly, the exploration of winter parks has not yet been in-depth, especially in cold winters. It cannot be ignored that even in cold climates, the need for residents to get close to nature remains unabated. It is still unknown whether winter parks in cold regions have psychological health restorative effects and their differences from non-winter ones. Thirdly, most studies did not differentiate the target group, and research methods may not be universally applicable. The cognitive process and affective feedback of older adults are different from those of young people, and their specificity should be considered in spatial representation and research methods. For example, older adults have higher requirements for environmental safety, social interaction (3), and are more inclined toward familiar environments (36). Based on these, this study focused on the recovery effect of older adults’ mental health, coordinating winter and non-winter, and intended to clarify the effects and processes of winter and non-winter park environments on the psychological health recovery of older adults. The former can help designers clarify the health recovery ability of winter and non-winter park environments, while the latter can help further understand the key points of optimizing park environments under seasonal differences.

Additionally, psychological processes have complex mechanisms, which are more evident in the restorative effects of mental health in park environments. After integrating existing research pathways, it can be found that environmental preferences are the result of environmental perception processes. If the discussion starts from physical environmental stimuli, it is necessary to discuss the spatial perception process of “sensation-perception-cognition” (37–40). Among this, perception refers to the interpretation of signals received by human organs through the heart and mind. Cognition is a process of information processing that includes sensation, perception, memory, thinking, imagination, and language. Considering that the entire psychological process is too complex to be clearly divided, and that existing research does not have a clear division of spatial constituent elements, characteristic elements, and perceptual elements, in order to avoid such problems, this study started with “perceptual environment” to discuss and simplify the psychological process. Furthermore, based on ABC theory of “cognition-affection-behavior” (41), this research shifted its focus to the discussion of different seasons, affective and behavioral directions, and constructed a “full path” for the restorative effect of mental health for older adults, in order to grasp the entire process of its implementation and positively intervene in park design practice from different stages.

## 2 Methodology and material

### 2.1 Site selection and data collection

**Figure 1:**
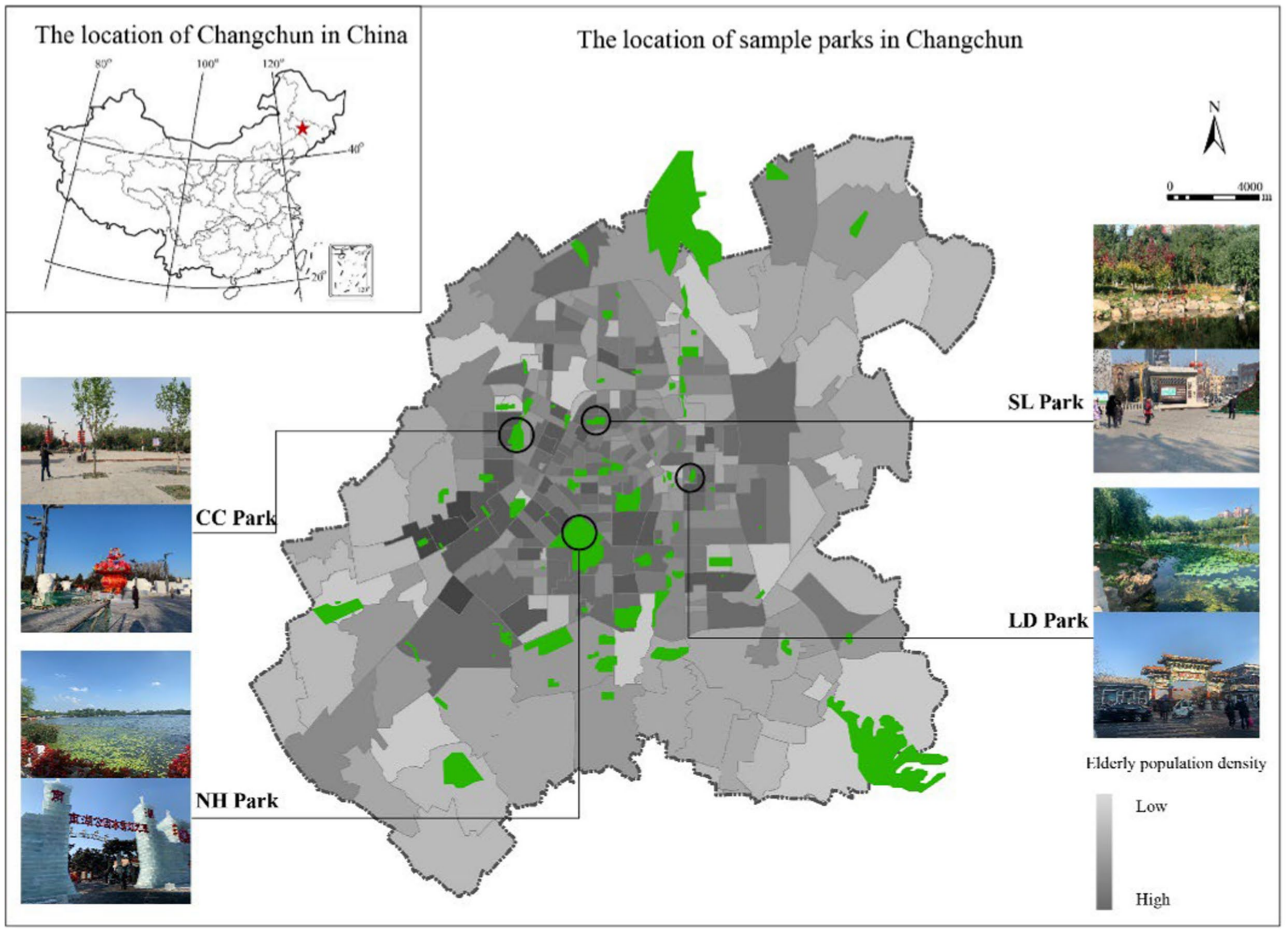
Sample parks location. Source: Modified from the data provided by Changchun Urban and Rural Planning and Design Institute.

This study took Changchun, a typical cold regional city, as an example. Changchun has distinct seasonal differences. Based on the definition of Chinese meteorological standards and monthly standards, combined with the actual situation in 2023, the average temperature in winter (November–April of the following year) is −9.3–1.8°C, and the average temperature in non-winter (May October) is 12.5–24.5°C. And NH Park, CC Park, SL Park, and LD Park as the research locations were selected (Figure 1). These above parks are all representative parks in Changchun, and are characterized by free admission, rich natural elements, convenient transportation, high density of surrounding communities, and high reachability and vitality of older adults.

The survey consists of three parts, divided into winter and non-winter periods. The first part is behavioral observation and semi-structured interviews. Based on the Mutually Exclusive Collectively Exhaustive (MECE) principle, 32 and 43 older adults were selected for in-depth interviews in winter and non-winter parks respectively, to understand the spatiotemporal behavior characteristics of them in different seasons of these parks, as well as the important spatial elements and features they consider important. The second part is a factor analysis survey. Based on the results of interviews, small-scale surveys were conducted, collecting 108 and 113 questionnaires in winter and non-winter respectively, to extract, classify, and name factors. The third part is the effect path survey, which is divided into pre survey and formal survey. Based on the pre survey results, the questionnaire items were revised. The winter formal survey was conducted from January to March 2022, and the non-winter formal survey was conducted from August to September 2022. Each park randomly selected 55–60 questionnaires, and the respondents obtained a total of 211 winter valid samples and 240 non-winter valid samples.

**Figure 2:**
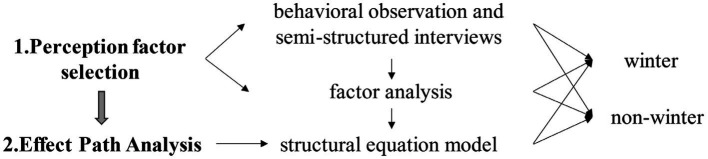
Research process and methods.

To sum up, observation, interview, SD, and factor analysis methods were used to determine observational and latent variables of perception factors, while the self-rated health approach was used to measure the restorative effects of mental health. Due to the unique characteristics for older adults, methods for obtaining physiological indicators through physiological testing equipment and common pressure methods are not applicable. Therefore, considering the cognitive style of older adults, discussing the restorative effects of psychological health through self-assessment based on psychological perception perspective is more feasible and practical. Unlike other objective measurement methods of physiological indicators, self-assessment of health is a subjective evaluation of the research object’s own health status (42), which is not stimulated or interfered with by the external environment and has high representativeness and sensitivity. Currently, this method is commonly used in many research reports of the World Health Organization to measure the quality of life and health status of older adults (43, 44). In terms of effect path analysis, this study used Structural Equation Modeling (SEM) for multiple mediation analysis, which quantifies observation variables to explore the motivators and pathways of the restorative effects of mental health for older adults in parks (Figure 2).

### 2.2 Path assumption

**Figure 3:**
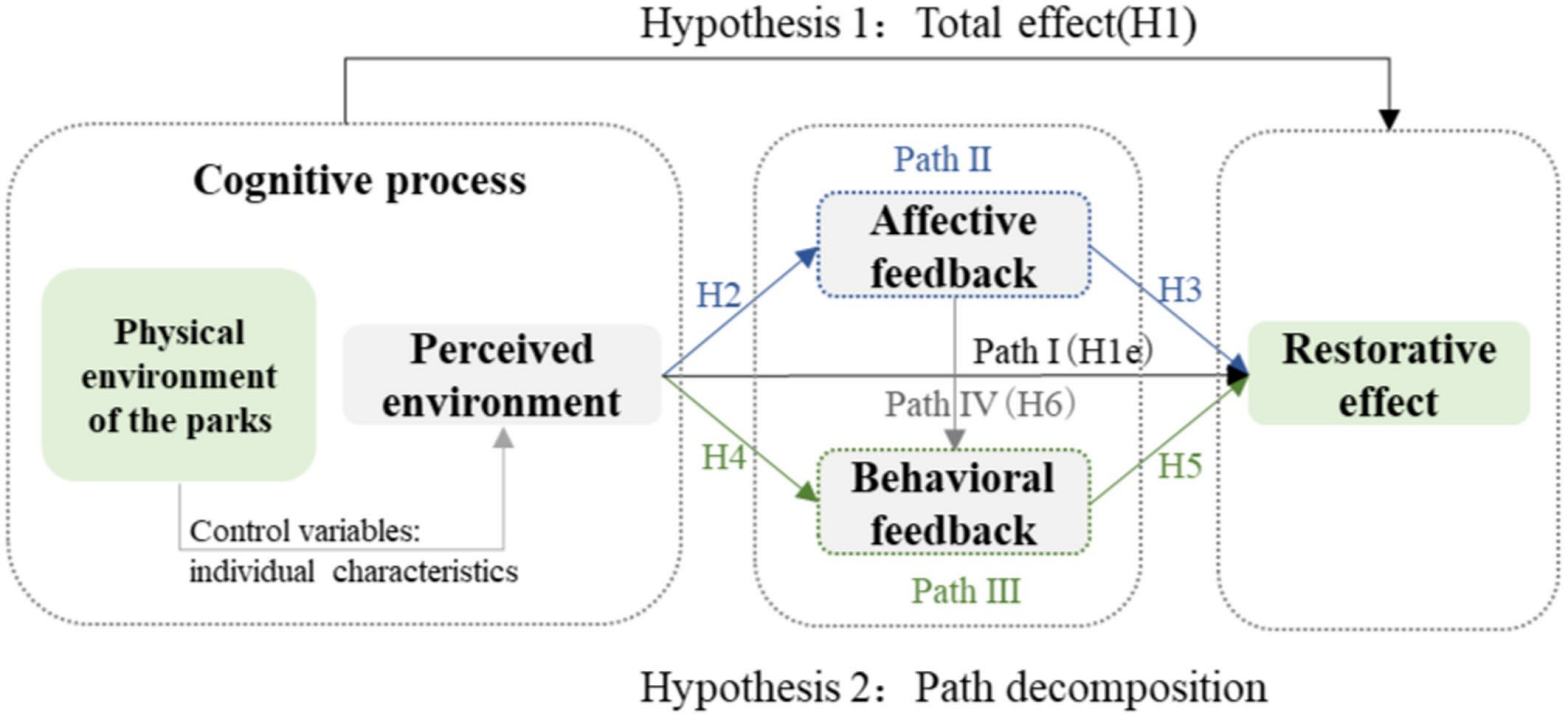
Research hypothesis.

Based on ABC theory, this study proposed two hypotheses (Figure 3): Hypothesis 1, corresponding to the overall effect, the specific content is: H1. Winter parks can significantly positively affect the restorative effect of older adults’ mental health. Hypothesis 2, corresponding to the decomposition of effect paths, specifically including: Path I (direct effect), H1e. Perceived environmental assessment can directly and significantly positively affect the restorative effect of mental health for older adults; Path II: H2. Perceived environmental assessment can significantly and positively affect the affective feedback for them in parks, and H3. Affective feedback can significantly and positively affect the restorative effect of older adults’ mental health; Path III: H4. Perceived environmental evaluation can significantly and positively affect the behavioral feedback for them in parks, and H5. Behavioral feedback can significantly and positively affect the restorative effect of mental health for them; Path IV: H6. Affective feedback can positively affect behavioral feedback. Additionally, this research assumed that the mediating pathways of “perceived environment → affective feedback → restorative effect,” “perceived environment → behavioral feedback → restorative effect,” and “perceived environment → affective feedback → behavioral feedback → restorative effect” all exist.

### 2.3 Variable settings

Firstly, based on the interview results, the study obtained the spatial composition and characteristic elements that older adults consider important in the park, and preliminarily classified the influencing factors into 16 in winter and 20 in non-winter. Then, it classified and named these influencing factors through Semantic Differential (SD) method and factor analysis method. The survey conducted reliability and validity tests, and then exploratory factor analysis (EFA) was used to extract 5 dimensions, with feature root values greater than 1. The variance interpretation rates of the five rotated factors in winter were 20.152, 19.824, 15.787, 14.803, and 14.148%, respectively. The cumulative variance interpretation rate after rotation was 84.71%. In non-winter, those were 18.826, 16.676, 12.694, 12.680, and 11.942%, and the cumulative variance interpretation rate after rotation was 72.817%. The results indicated that these factors are evenly distributed and can explain most of the differences in variables. And then, factors were named based on the properties and characteristics of each dimension, and were divided into five categories: (I) sense of security factors, (II) sense of comfort factors, (III) sense of interest factors, (IV) sense of convenience factors, and (V) sense of belonging factors (Table 1). These five types of factors are also similar to Meta’s proposal based on the classic concept that good public spaces should have characteristics (45), which indirectly confirms the rationality of the classification and naming of perceptual environmental evaluation factors.

**Table 1:** Load factor after rotation.

Dimension name	Winter	Factor loading					Non-winter	Factor loading				
		1	2	3	4	5		1	2	3	4	5
I sense of security factors	A1 Flat paving			0.465			A1 Flat paving				0.643	
	A2 Reasonable height difference			0.572			A2 Reasonable height difference				0.819	
	A3 Reasonable lighting facility settings			0.896			A3 Reasonable lighting facility settings				0.784	
	A4 Clear direction			0.831			A4 Clear direction				0.498	
II sense of comfort factors	S1 Pleasant acoustic environment		0.707				S1 Pleasant acoustic environment					0.848
	S2 Fresh air		0.778				S2 Fresh air					0.742
	S3 Comfortable leisure facilities		0.773				S3 Comfortable leisure facilities					0.614
							S4 Good shading conditions					0.643
III sense of interest factors	Q1 Rich plant hierarchy and evergreen plants					0.684	Q1 Rich plant species	0.705				
	Q2 Available water space					0.872	Q2 Rich plant colors	0.788				
	Q3 Rich structures, landscape sketches or ice and snow sculptures					0.641	Q3 Rich plant hierarchy	0.808				
							Q4 Beautiful waterscape	0.769				
							Q5 Rich structures and landscape sketches	0.678				
IV sense of convenience factors	B1 Good functional space reachability	0.889					B1 Good functional space reachability		0.635			
	B2 Adequate activity facilities and space	0.889					B2 Adequate activity facilities and space		0.865			
	B3 Frequently updated and maintained activity facilities	0.745					B3 Adequate leisure facilities		0.613			
							B4 Frequently updated and maintained various facilities		0.606			
V sense of belonging factors	G1 Easily recognized park features				0.861		G1 Easily recognized park features			0.566		
	G2 Good cultural deposition				0.786		G2 Good cultural deposition			0.709		
	G3 Rich activity forms				0.701		G3 Rich activity forms			0.764		

On these grounds, this study used these five types of factors as latent variables for perceived environmental evaluation, affective and behavioral feedback as mediating variables, older adults’ individual characteristics as control variables, and the restorative effects as dependent variables to establish a full path model. In the affective feedback stage, based on the pathway of “environmental preference → place attachment → restorative effect,” place attachment is usually divided into two dimensions: place dependence (PD) and place identity (PI) (46). PD is a functional attachment that focuses on the function of a place to meet individual needs, emphasizing an individual’s sense of belonging to the place. PI is an affective attachment that leans more toward the internal emotional connections between individuals and places, emphasizing the shared values, attitudes, thoughts, beliefs, etc. Some studies have also shown that place attachment mainly affects people’s attitudes and behaviors toward the environment through the mediating role of place identity (47). Therefore, E1 Preference (degree of liking for the environment), E2 Dependence (degree of belonging and functional attachment to the environment), and E2 Identity (degree of identification and affective attachment to the environment) were selected as the observation variables for affective feedback. In the behavioral feedback stage, based on the distinction between function and exercise intensity, a reasonable and observable classification method with unified standards was selected, which includes Moderate and Vigorous Physical Activity (MVPA), and Low Physical Activity (LPA) (48). Moreover, based on behavioral observation results, the study divided MVPA into M1 Walking and running behaviors (fast walking, jogging, etc.), M2 Fitness behaviors (Tai Chi, equipment sports, ice skating, etc.), M3 Recreational behaviors (square dance, etc.), and LPA into L1 Group leisure behaviors (playing cards, playing chess, etc.) and L2 Individual leisure behaviors (walking dogs, resting, etc.).

The observation of restorative effects was conducted through a self-assessment approach, drawing on the revised ROS scale by Kaplan et al. (12), and the World Health Organization’s Physical and Mental Health Scale (WHO-5). Six items were formed, including R1—relieving stress, R2—restoring attention, R3—improving emotions, R4—reducing loneliness, R5—increasing energy, and R6—eliminating fatigue and improving sleep quality. Each item of this survey was scored by the Likert scale 1–5 points. At the end, the collection of information on older adults themselves was added, including gender, age, frequency of visiting parks, and length of stay time.

## 3 Results

### 3.1 Descriptive statistics

211 and 240 valid sample data were obtained in winter and non-winter, respectively (Table 2). In the winter sample data, the proportion of older adults under 70 years old was relatively large, accounting for 60.62%; female accounted for 63.03%; the majority of older adults visited the parks 2–4 times a week, being 46.44%; 74.41% of them stayed in the parks for less than 2 h. In non-winter, female accounted for 62.92%; 42.92 and 32.91% were, respectively, aged 61–69 and > 70; 83.34% older adults visited the parks more than twice a week; and 40.42 and 35.00% of samples stayed in the parks for 1–2 h and more, respectively.

**Table 2:** Composition of survey samples.

			Winter		Non-winter	
			*N*	%	*N*	%
Gender	Female		133	63.03	151	62.92
	Male		78	36.97	89	37.08
Age	55–60		56	26.54	58	24.17
	61–69		93	44.08	103	42.92
	>70		62	29.38	79	32.91
Frequency of visiting park	Once a week or less		46	21.80	40	16.66
	Twice to four times a week		98	46.44	112	46.67
	More than four times a week		67	31.76	88	36.67
Length of stay time	Less than 1 h		76	36.02	59	24.58
	1–2 h		81	38.39	97	40.42
	Over 2 h		54	25.59	84	35.00

### 3.2 Reliability and validity test

After SPSS and AMOS analysis, the overall reliability and validity of the pathway study were obtained. Cronbach α for winter and non-winter data showed that both overall and partial reliability were good. The data also passed KMO and Bartlett’s tests, and the KMO values were both greater than 0.9, indicating that it can be further analyzed. Then, this study conducted confirmatory factor analysis (CFA) to test the rationality of each sub item setting within the model (Table 3). Firstly, standard load coefficient values are usually used to represent the correlation between factors and analysis items (measurement items). The results showed that the standard load coefficients were all greater than 0.7 and significant, indicating a strong correlation between observed variables and latent variables; secondly, Mean Variance Extraction (AVE) and Combined Reliability (CR) values were used to test the aggregated validity of different dimensions. AVE were all greater than 0.5 and CR were all greater than 0.7, indicating that the aggregated validity of the model was high; in addition, the discriminant validity test was conducted using the correlation matrix between factors, and the model still had good discriminant validity.

**Table 3:** Reliability and validity test of winter and non-winter models.

Dimension	Latent variable	Winter			Non-winter		
		Cronbach α	AVE	CR	Cronbach α	AVE	CR
Perceived Environmental Stage	F1 sense of security factor	0.928	0.770	0.930	0.915	0.739	0.918
	F2 sense of comfort factors	0.939	0.839	0.940	0.890	0.626	0.893
	F3 sense of interest factors	0.847	0.665	0.856	0.851	0.545	0.856
	F4 sense of convenience factors	0.919	0.801	0.923	0.912	0.720	0.911
	F5 sense of belonging factors	0.841	0.647	0.845	0.863	0.700	0.872
Affective feedback stage	F6 affective feedback	0.894	0.741	0.895	0.725	0.580	0.734
Behavioral feedback stage	F7 MVPA feedback	0.831	0.632	0.837	0.874	0.714	0.882
	F8 LPA feedback	0.836	0.730	0.843	0.696	0.553	0.711
Recovery stage	F9 restorative effects	0.906	0.630	0.910	0.785	0.510	0.805

### 3.3 Model fitting results

**Figure 4:**
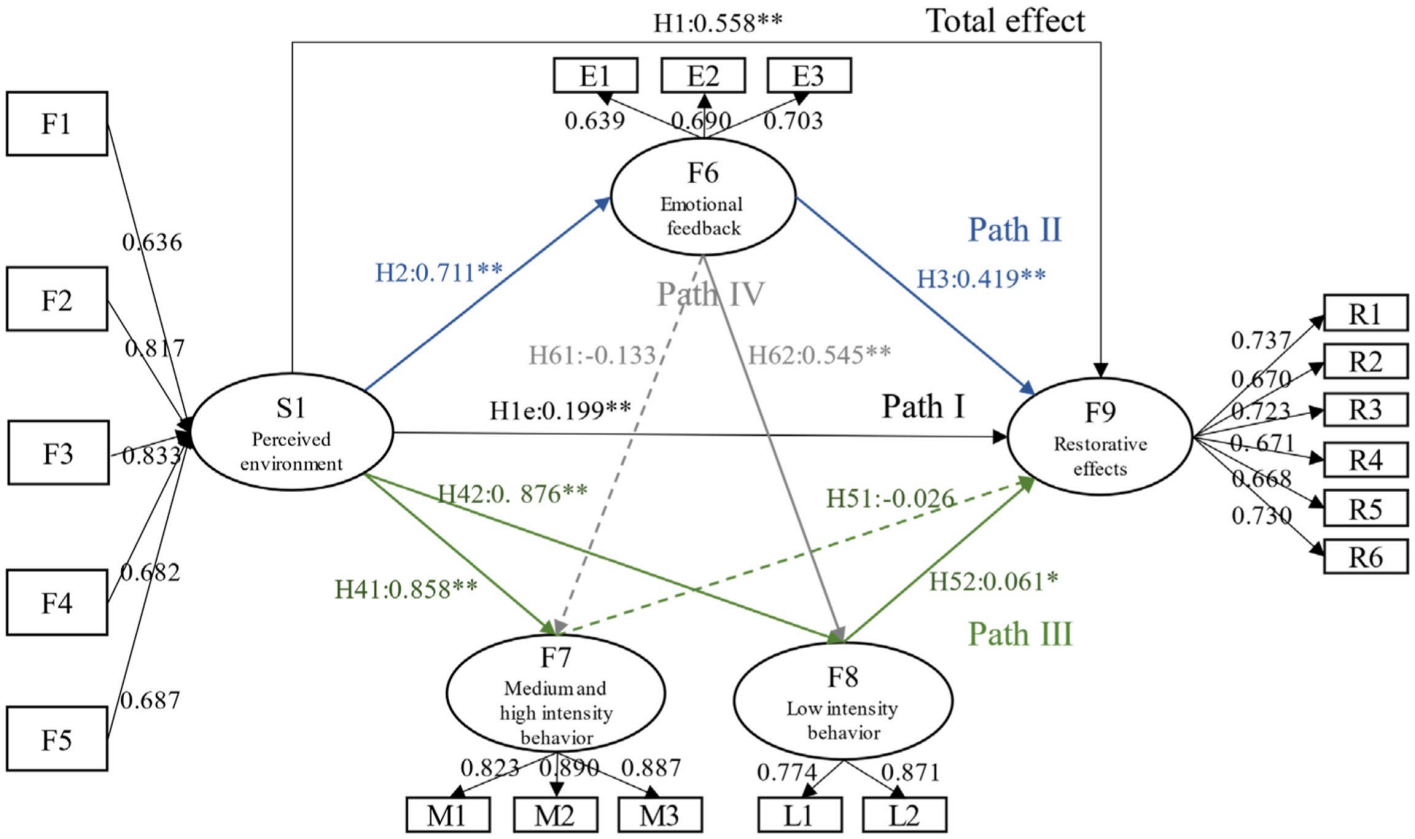
Model and path coefficient of the restorative effects in winter parks.

**Figure 5:**
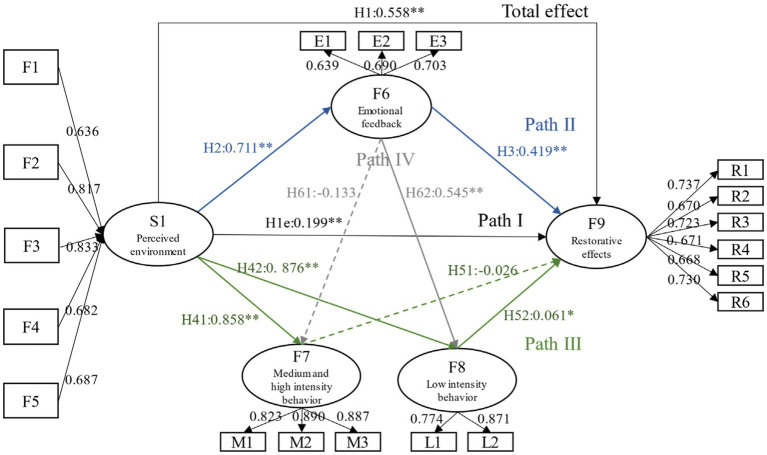
Model and path coefficient of the restorative effects of in non-winter parks.

To make the research results more concise, this study firstly used the average of specific observation variables to measure five perceived environmental factors, and simultaneously formed S1 perceived environmental evaluation by combining the five factors. Then separate F7 and F8 to form H41, H42, H51, H52, and H61, H62 assumptions. Including: H41. F7 can significantly positively affect F9; H42. F8 can significantly positively affect F9; H51. S1 can significantly positively affect F7; H52. S1 can significantly positively affect F8; H61. F6 can significantly positively affect F7; and H62. F6 can significantly positively affect F8. The results of various important fitting indicators have reached a good or acceptable level, proving the usability of the model (49–51) (Table 4). The regression results of the model were shown in Figures 4, 5. As shown in the results, in winter, H1e and H52 were not valid, and the overall effect, path 2, and path 4 assumptions were also valid; in non-winter, H51 and H61 were not valid, and all other assumptions were valid.

**Table 4:** List of important fitting indexes of non-winter and winter model.

	χ2/*df*	GFI	RMSEA	RMR	CFI	NFI	NNFI
Acceptable range	<3	>0.7	<0.10	<0.05	>0.7	>0.7	>0.7
Winter	2.914	0.754	0.094	0.026	0.858	0.805	0.842
Non-winter	2.426	0.769	0.077	0.011	0.874	0.806	0.857

Furthermore, the bootstrap (sample = 5,000) automatic sampling method was used to test mesomeric effect, and the results were shown in Table 5. If the confidence range of bootstrap does not contain 0, it indicates that mediating effect exists; if the direct effect does not exist and the mesomeric effect exists, it is completely mediated, otherwise it is partially mediated (52). The results illustrated that in winter, affective feedback and MVPA feedback played a completely mediating role in the recovery effect of older adults, and the mesomeric effect of affective feedback was slightly higher than that of behavioral feedback path; and the chain mesomeric effect of “perceived environment → affective feedback → MVPA feedback → restorative effect” existed. In non-winter, affective feedback and LPA feedback played a partial mediating role, and the effect value of the former was still higher than that of the latter; the chain mediation effect of “perceived environment → affective feedback → LPA feedback → restorative effect” existed.

**Table 5:** Intermediary effect test.

		Path	Effect	Boot SE	BootLLCI	BootULCI	*Z*	*p*	Conclusion
Winter	Path II	S1 ⇒ F6 ⇒ F9	0.346	0.059	0.262	0.446	5.888	0.003	Supported
	Path III	S1 ⇒ F7 ⇒ F9	0.110	0.048	0.022	0.209	2.303	0.021	Supported
		S1 ⇒ F8 ⇒ F9	0.010	0.025	−0.037	0.063	0.396	0.692	Not supported
	Path IV	S1 ⇒ F6 ⇒ F7 ⇒ F9	0.021	0.016	0.002	0.050	1.321	0.094	Supported
		S1 ⇒ F6 ⇒ F8 ⇒ F9	0.009	0.027	−0.036	0.055	0.332	0.681	Not supported
Non-winter	Path II	S1 ⇒ F6 ⇒ F9	0.298	0.060	0.310	0.546	4.940	0.000	Supported
	Path III	S1 ⇒ F7 ⇒ F9	−0.022	0.051	−0.130	0.043	−0.050	0.577	Not supported
		S1 ⇒ F8 ⇒ F9	0.053	0.049	−0.017	0.180	1.083	0.279	Supported
	Path IV	S1 ⇒ F6 ⇒ F7 ⇒ F9	0.002	0.006	−0.008	0.019	0.377	0.706	Not supported
		S1 ⇒ F6 ⇒ F8 ⇒ F9	0.024	0.021	−0.008	0.077	1.119	0.263	Supported

Based on previous interviews and surveys, it was found that the frequency and duration of older adults visiting parks in winter were lower than those in non-winter. Overall, parks had better restorative effects on older adults aged 61–69 and > 70. The higher the frequency of visiting parks and the longer the duration of stay time, the better the affective feedback and the stronger the restorative effect. This was consistent with the conclusion of a case study in Netherlands that the higher the frequency of people visiting urban parks, the closer their connection and attachment to them (53).

In winter, influenced by extreme weather conditions, there were significant differences in spatial perception and temporal spatial behavior among older adults in the parks, especially among women, middle—aged and the very older adults. For example, roads covered with ice and snow can reduce the sense of security for older adults, thus limit their travel possibilities and activity duration. The cold and single landscape in winter can also weaken its visual experience, reducing the frequency of travel to a certain extent. However, interesting and storytelling ice and snow landscapes can enhance spatial attractiveness, promote their preference and attachment to the environment, and thus increase the frequency and duration of visits. Due to biophilia and fitness goals, about one-third of older adults still exercised “in all weathers.” This type of older adults’ activities in the parks were usually concentrated between 9:00 am to 11:00 am and after 18:00 pm. Daytime activities were mainly about walking, dancing, and equipment sports, while evening activities were mainly focused on dancing. As for non-winter, the park environment was vibrant and shaded by green trees, and the probability of older adults engaging in low-intensity behavior was significantly increased, mainly including sightseeing, rest, taking care of grandchildren, playing cards, and so on. In addition, in terms of spatial distribution, winter had more obvious clustering, and the hot spot spaces were usually adjacent to the entrance and exit and the main road in the parks; in non-winter, it appeared to be more dispersed and built-in, with an increased likelihood of internal space being explored. In summary, the relevant results can indirectly confirm the importance of discussing the restorative effects of parks in different seasons.

## 4 Discussion

### 4.1 Discussion on the total effect under seasonal differences

H1 was to verify the total effect of the parks’ restorative effect on older adults mental health. The results indicated that even in winter, parks still had restorative effects, with the total effect coefficient of 0.479; the total effect coefficient of non-winter was 0.558, which was higher than that in winter.

Speaking separately, comparing the correlation coefficients of five types of perceived environmental evaluation factors on restorative effects in winter, the ranking is: F4 sense of convenience factors (0.915) > F5 sense of belonging factors (0.898) > F1 sense of security factors (0.687) > F3 sense of interest factors (0.626) > F2 sense of comfort factors (0.603). (1) Among them, the most important purpose for older adults to visit parks in winter was to exercise, so the coefficient of convenience was the highest in terms of whether the venue was easy to reach, followed by the adequacy of fitness facilities and space. The phenomenon that older adults usually chose to exercise in spaces closer to entrances and exits in the survey may provide some explanation. (2) Among the factors of sense of belonging, the most important was rich forms of activities, followed by whether the park is distinctive. Although each sample park has its representative characteristics, it is particularly evident in LD Park. Many older adults believed that parks with bright colors and ethnic characteristics will be more attractive, and they preferred a lively and familiar atmosphere, which will encourage them to linger and bring more companions. (3) Regarding the factors of sense of security, it was particularly important to have a reasonable height difference design and a smooth and anti-slip paving. In the interview, older adults expressed that it is inevitable to accumulate ice and snow in winter, and pedestrians wear heavy clothes, which makes it very difficult to take steps and other actions. Falling has become one of the important threats to older adults’ health, so it is very important to ensure that the floor is not slippery. (4) As for sense of interest, the factors with higher path coefficients were the available water space, rich plant layers and evergreen plants. This was consistent with Kaplan’s results, as even in winter, the presence of water and plants is quite important. This also echoed other research findings, such as the fact that a snowless vegetation environment on streets in winter can help alleviate people’s mental fatigue (54); and winter entertainment activities in snowy forests also have psychological recovery effects (55, 56). In the survey, ice and snow sports relying on water were more attractive to older adults men. Besides that, ice sculpture exhibitions similar to NH Park and snow sculpture activities in CC Park can enhance their sense of participation and novelty. (5) About sense of comfort factors, the most important was fresh air. Most older adults said that “exercising every day is to breathe fresh air, otherwise I will feel suffocated.” Followed by the sound environment, they said that “if there is music and songs, the mood will be better.” And due to the winter climate, they rarely sited and rested outdoors, so the comfort of rest facilities ranked last.

Unlike winter, the ranking of perceived environmental factors in non-winter was: F3 sense of interest factors (0.833) > F2 sense of comfort factors (0.817) > F5 sense of belonging factors (0.687) > F4 sense of convenience factors (0.682) > F1 sense of security factors (0.636). Firstly, the most important elements were plant and water in F3. Green plants can relax the human nervous system, make the body calm, comfortable, and vigorous, which is beneficial for promoting long-term psychological health of older residents (57); the presence of water can enhance the alertness of older adults in parks (58). So that a plant landscape with rich colors, levels, and types, as well as beautiful water features can bring positive visual experience and potential for restoration. It is worth mentioning that older adults showed a high preference for brightly colored landscape. Secondly, the most important comfort factors were fresh air and good shading conditions. The former was similar to the winter results, where “breathing fresh air” is like “exchanging blood” for older adults, and is an important way to rejuvenate oneself, which is similar to “forest bathing” (59). Thirdly, in the sense of belonging factors, the order of each observation variable was the same as in winter, and a lively and familiar atmosphere had more potential for recovery. Fourthly, among the convenience factors, the setting of rest facilities had the highest path coefficient, which was different from winter. This may be related to the behavioral content of older adults in non-winter. Adequate rest facilities can not only provide them with a place to rest, but also provide them with social space. Finally, in F1, the most important still lied in the rationality of vertical and paving design. This was most evident in NH Park, as it is equipped with a plastic walkway, which can enhance the comfort and safety of sports with its cushioning and anti-slip performance.

### 4.2 Discussion on each effect path under seasonal differences

#### 4.2.1 Discussion on Path I under seasonal differences

Path I, namely the direct impact of perceived environmental assessment on the restorative effect of older adults’ mental health. It was indicated that perceived environmental assessment was not the direct antecedent of restorative effects in winter. This was not entirely consistent with existing research conclusions (60), which may be caused by different climate regions and seasons. One of the possible reasons is that the winter landscape in cold regions is single, which cannot provide good visual stimulation and comfortable activity space, resulting in a decrease in the overall subjective perception of environmental evaluation results, and thus the direct effect path cannot be established; another reason may be that although the direct effect of perceived environmental evaluation on restorative effects was negative, the total effect reached 0.479, indicating that winter parks may exert restorative effects more through affective and behavioral feedback.

In non-winter, the direct effect of “perceived environment → restorative effect” was significant, indicating that different elements and features of space can directly have positive restorative effects. Compared to winter, the non-winter park environment is lush, more vibrant, and able to enhance the subjective perception of space by older adults. This is similar to the conclusion in ART theory, where a good space can provide visitors with feelings of being away (getting rid of their current exhausted life state), fascination (naturally captivating), compatibility (providing conditions that are consistent with people’s preferences and activities), and extent (its richness and coherence allowing them to fully explore) (61). Consistent with other existing research findings, urban green spaces have a positive impact on visitors’ emotions and arousal (9); natural environment can enable people to experience more positive emotions, including satisfaction, happiness, and peace, as well as alleviate negative emotions such as anxiety, fear, and anger (10).

#### 4.2.2 Discussion on Path II under seasonal differences

Path II assumed that the park environment affects the restorative effect of mental health through affective feedback, which was simply the effect path of “perceived environment → affective feedback → restorative effect.” This path contained assumptions about H2 and H3. Both winter and non-winter results supported the relevant assumptions in Path II and confirmed the existence of mediation pathway, which was in consensus with the existing research conclusions (62). Among them, the order of the observed variables in affective feedback is: identification > dependence > preference. The possible reason is that identity is a higher-order affective feedback compared to preference and dependence, and residents’ functional dependence on space has a positive impact on the formation of affective attachment. Place dependence, as an intermediary element of place identity, can affect people’s attitudes and behaviors toward the environment (47), thus having a greater impact on restorative effects.

Further connections have been established between affective feedback and five types of perceptual factors in winter and non-winter, respectively. It was found that both in winter and non-winter, the factors of sense of belonging and sense of interest ranked high in terms of importance. Affections may be more about stimulating feedback through visual dimensions. In terms of spatial cognition methods and approaches, it can be divided into “scientific cognition” and “experiential cognition,” as well as “experiential spatial cognition” and “constructive spatial cognition.” Scientific and experiential cognition is objective and exclusive, which is the result of abstraction and rationalization of space directly through the senses; Empirical and constructive approaches require the affective participation of cognitive subjects (35). The long-term experience and accumulation of experience make the subjective cognitive role of older adults more prominent. As a result, older adults have a greater preference for familiar environments and stronger feedback on situational triggered affective experiences.

#### 4.2.3 Discussion on Path III under seasonal differences

Path III contained the contents of H4 and H5. The results confirmed that the park environment exerted restorative effects by promoting older adults’ behavior, which was consistent with the conclusion in existing studies that behavior can promote restorative effects (58). Differently, the results showed that H52 in winter was not valid, and H51 in non-winter was not. In other words, the restorative effect of the winter park was achieved through mediating pathways that promote older adults MVPA; while in non-winter, it was achieved by promoting LPA.

In winter, due to the influence of climate, older adults must “move” in the park to achieve the goal of physical fitness, so they mainly engage in MVPA. Among such behaviors, older adults tended to engage in more recreational activities, such as yangko dancing, followed by fitness activities such as skating, equipment sports and taichi, and finally, fast walking and jogging. About LPA instead, older adults tended to engage in group leisure activities, such as playing chess, playing cards, etc., while individual leisure activities such as walking and walking dogs came in second place. Correspondingly, in non-winter, older adults were more likely to engage in low-intensity behaviors compared, especially in sightseeing, resting, and other behaviors; among MVPA, equipment sports and dance exercises were the majority.

Similarly, the relationships between different perceived environmental factors and behaviors of different intensities have also been further explored. It was found that there are certain differences in the ranking of the impact of five types of perceived environmental evaluation factors on behaviors of different intensities. On the one side, the top two perceived environmental evaluation factors that affected MVPA were F4 sense of convenience factors and F1 sense of security factors. The main reason may be that when older adults engage in high-intensity behaviors, they pay more attention to the safety of the site, whether there are potential hazards causing physical damage. The convenience and accessibility of the place, as well as the adequacy of facilities and space, are also key considerations, while other factors will only help them improve the enthusiasm and sustainability to a certain extent. On the other side, the main factors that affected LPA were: F5 sense of belonging and F3 sense of interest factors. The possible reason is that low intensity behaviors have relatively small movement rates and amplitudes, and older adults have a stronger ability to perceive the impact of visual dimensions in space. They have a higher demand for bright colored decorations and structures, soundscapes, and odors.

#### 4.2.4 Discussion on Path IV under seasonal differences

Path IV was about the relationship between affective feedback and behavioral feedback. In winter, both H61 and H62 were valid, indicating that affective feedback can positively affect both MVPA feedback and LPA feedback, with a greater coefficient of influence on LPA (0.512). For example, architectural spaces with cultural heritage or regional characteristics are more likely to stimulate the preferences and attachment of older adults, thereby promoting the development of group and individual leisure behaviors, while a lively atmosphere will attract more older people to dance or stop to observe. In the test of chain mesomeric effect, it found that there was a path of “perceived environment → affective feedback → MVPA feedback → restorative effect.”

As for non-winter, H61 was not established, and affective feedback from older adults people can only positively affect low-intensity behavior. The chain mediation path of “perceived environment → affective feedback → LPA feedback → restorative effect” had been proved. These results all validated the standard learning hierarchy model in attitude theory (63), which states that the attitude (Cognition) of older adults in the park directly affects their feelings or emotions toward the park (Affection), which in turn affects their behavior (Behavior), and then affects the acquisition of restorative effects. The higher the perceived environmental quality of a space, the higher the level of preference and attachment among older adults, the more willing they are to engage in various behaviors in this space, and thus the better the recovery effect of the space on them.

## 5 Conclusion

On the whole, this study discussed winter and non-winter separately, constructed the “full path” of the restorative effects on older adults’ mental health in the parks, and verified the six assumptions and mesomeric effect of its split. The results showed that: (1) both winter and non-winter parks had restorative effects on older adults’ mental health, and the overall effect coefficient was slightly higher in non-winter. (2) In winter, H1e and H52 were not be supported, and perceived environment evaluation was not the direct antecedent of restorative effects. Whereas, perceived environment played a role through affective feedback and MVPA feedback, and a chain mediated path of “perceived environment → affective feedback → MVPA feedback → restorative effect” existed. (3) In non-winter, H51 and H61 were not valid, and the restorative effects were achieved through direct and indirect effects. A good perceptual environment can directly and positively affect the acquisition of restorative effects. The indirect paths of “perceived environment → affective feedback → restorative effect,” “perceived environment → LPA feedback → restorative effect,” and “perceived environment → emotional feedback → LPA feedback → restorative effect” all existed.

Based on the above path decomposition, park optimization can be divided into two approaches: “affective inducing” and “behavior promoting.” The “affective inducing” parks should focus on creating its own regional cultural characteristics and a lively atmosphere based on social activity organization. Therefore, in planning and design, on the one hand, attention should be paid to the expression of narrative landscape structure, connecting story lines according to different landscape situations, bringing older adults into a positive “nostalgic” complex (64, 65), and enhancing their sense of identification and dependence on the environment. The “nostalgia” therapy has been proven to awaken local attachment for older adults and can play a role in psychological recovery and healing (66). On the other hand, in terms of detail design, elements with regional characteristics can be appropriately used to stimulate the visual experience of older adults. Landscape sketches with local flavor that can be appropriately added to create a familiar and nostalgic atmosphere in non-winter. Classical landscapes can also be added to enhance their additional value. In winter, the display of ice and snow sculptures and the organization of characteristic activities can enhance the sense of participation of older adults in parks.

The “behavior promoting” parks should differentiate various sports intensities, dividing them into high-energy areas and leisure areas. In winter, the high-energy zone mainly includes path space, water space, instrument space, and square space, while in non-winter, it mainly includes path space, square space, and instrument space. The leisure area mainly involves path space, structure space, and vegetation space. High-energy areas should prioritize the enhancement of convenience and security, while leisure areas should enhance the creation of a sense of interest and belonging. It should be noted separately that the path space, as an important spatial carrier for older adults’ sports in the parks, should prioritize the optimization of security factors, and should be zoned for planning and strengthened guidance. For example, in winter, the path space should be cleared of snow as soon as possible, and a plastic dual color track with good elasticity should be selected. Running and walking areas should be divided to prevent mutual interference of different behaviors and improve the safety and comfort of sports. Plants on both sides can add colorful decorations to enhance the fun of the space. In addition, for other functional spaces, in winter, on the premise of timely clearing of snow, the first priority should be to coordinate the size of the space and the adequacy and maintenance of facilities. Semi indoor fitness facilities, item placement facilities, etc. can also be appropriately added to reflect humanistic care in details. Winter themed activities can also be organized with communities, such as ice sculpture and snow sculpture exhibition activities, to enhance spatial attractiveness and participation. In non-winter, the most important thing is to improve the color, form, and layering of plants, and create a pleasant visual, auditory, and odor environment.

Due to the limitations of special climate and special group, the sample acquisition and research results of this study had certain limitations. However, it can still provide a complete exploration logic for related field research and provide certain references for spatial positive intervention at different stages. Future research can be based on this to conduct more in-depth experimental studies, further deepening the different psychological processes of different groups. Thus, intervention design methods corresponding to different psychological processes can be formed.

## Data availability statement

The datasets presented in this article are not readily available because due to the nature of this research, participants of this study did not agree for their data to be shared publicly, so supporting data is not available. Requests to access the datasets should be directed to TY, ytjhit@126.com.

## Ethics statement

For the studies involving humans because ethical review and approval was not required for the study on human participants in accordance with the local legislation and institutional requirements. The studies were conducted in accordance with the local legislation and institutional requirements. The ethics committee/institutional review board also waived the requirement of written informed consent for participation from the participants or the participants’ legal guardians/next of kin because written informed consent from the (patients/participants OR patients/participants legal guardian/next of kin) was not required to participate in this study in accordance with the national legislation and the institutional requirements.

## Author contributions

TY: Writing – review & editing, Writing – original draft, Supervision, Software, Methodology, Investigation, Formal analysis, Data curation, Conceptualization. HL: Writing – review & editing, Supervision, Resources, Funding acquisition, Conceptualization. ZY: Writing – review & editing, Investigation, Supervision.
